# Experimental filtering effect on the daylight operation of a free-space quantum key distribution

**DOI:** 10.1038/s41598-018-33699-y

**Published:** 2018-10-17

**Authors:** Heasin Ko, Kap-Joong Kim, Joong-Seon Choe, Byung-Seok Choi, Jong-Hoi Kim, Yongsoon Baek, Chun Ju Youn

**Affiliations:** 1 Photonic/Wireless Convergence Components Research Division, Electronics and Telecommunications Research Institute, Daejeon, 34129 South Korea; 2 School of Advanced Device Technology, University of Science & Technology, Daejeon, 34113 South Korea

## Abstract

One of the challenges of implementing free-space quantum key distribution (QKD) systems working in daylight is to remove unwanted background noise photons from sunlight. Elaborate elimination of background photons in the spectral, temporal, and spatial domains is an indispensable requirement to decrease the quantum bit error rate (QBER), which guarantees the security of the systems. However, quantitative effects of different filtering techniques and performance optimization in terms of the secure key rate have not been investigated. In this study, we quantitatively analyze how the performance of the QBER and the key rates changes for different combinations of filtering techniques in a free-space BB84 QKD system in daylight. Moreover, we optimize the conditions of filtering techniques in order to obtain the maximum secure key rate.

## Introduction

Free-space quantum key distribution (QKD) provides the availability of unconditionally secure communication for moving platforms and places without fiber-based infrastructure^1–6^. Even for the urban areas where fiber-optic communication network is already deployed, a free-space QKD can be sometimes desirable rather than a fiber-based QKD in terms of achieving superior performance. As a fiber-based QKD system shares fiber links with the other optical communication, the performance of the fiber-based QKD can be significantly degraded due to a substantial amount of scattered lights. Such lights come from the laser sources for the other fiber-optic communication whose power is several orders of magnitude stronger than those used for the QKD system^7^. Moreover, the deployment of an additional fiber network with a dedicated fiber link for a QKD system may not be a satisfactory option because it is very cost-ineffective in many practical situations.

In daylight, eliminating scattered noise photons from sunlight radiation is extremely important for the performance of a free-space QKD system. There are several reports of successful demonstration of QKD systems under daylight conditions^3–6^. They utilized multiple noise filtering techniques in the spectral, temporal, and spatial domains to effectively eliminate noise photons from sunlight. However, the effects of each filtering technique and performance comparisons with different combinations of filtering techniques have not been sufficiently investigated. Moreover, the detail system issues closely related to the filtering techniques, which are highly important for practical system implementation, have never been examined. For example, issues, such as optimized temporal filtering window in terms of the secure key rate and fluctuations of the real-time key generation rate for different sizes of the spatial field of view (FOV), have never been quantitatively covered in the previous studies.

In this study, we investigate how background noise photons are reduced as the filtering techniques in the spectral, temporal, and spatial domains are installed with several different combinations in a polarization-based BB84^8^ QKD system operating over a free-space distance of 275 m in daylight. First, we experimentally measure the amount of noise photons and the system performance with a baseline filtering setup at night. Second, we repeat the experiment in daylight to show an increased amount of noise photons and the corresponding performance degradation of the QKD system. Third, we experimentally demonstrate the performance improvement of the QKD system as we utilize more elaborate filtering techniques. Finally, we further discuss some system issues related to filtering techniques for performance optimization of QKD systems in daylight.

## Experimental Setup

**Figure 1:**
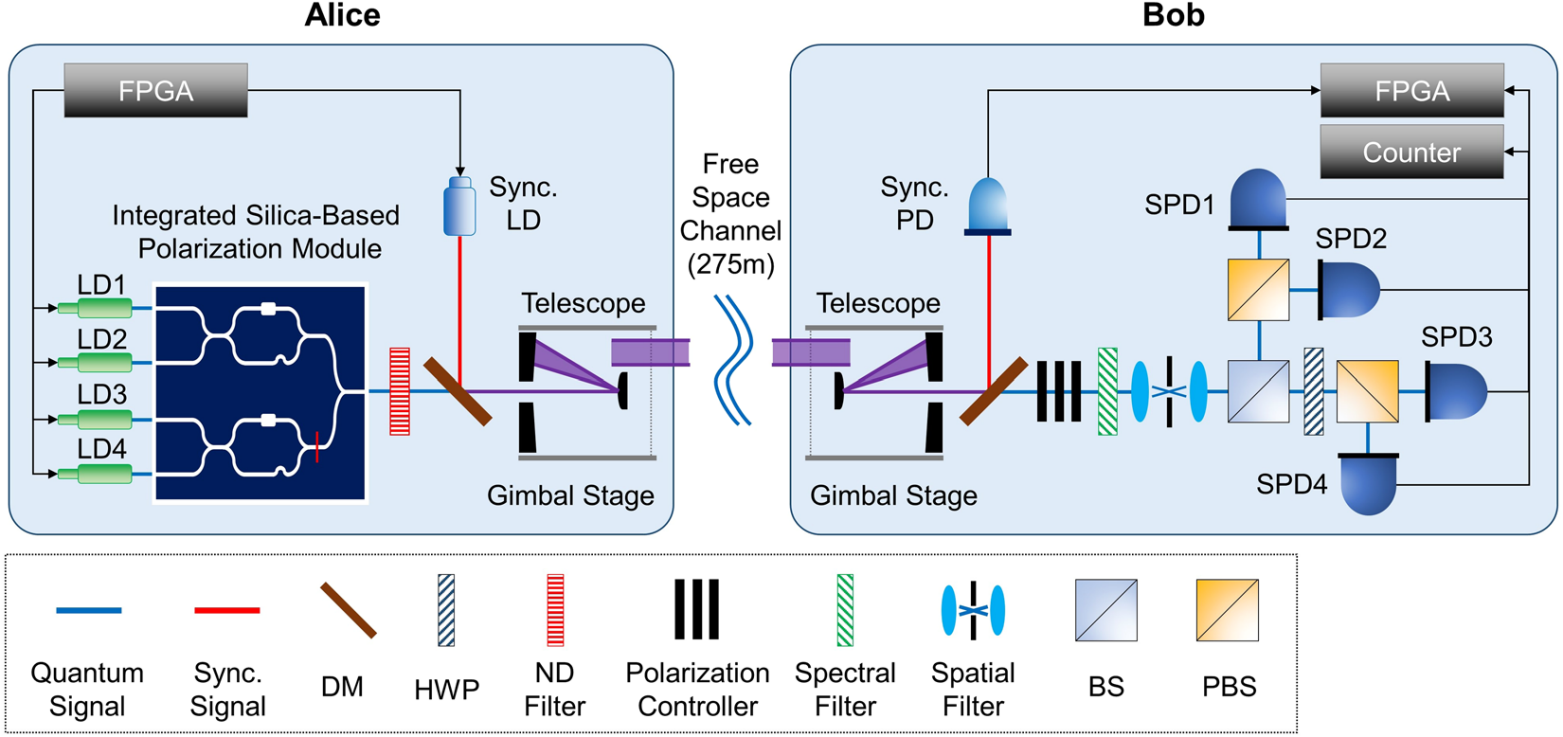
Experimental setup for measuring the effect of filtering techniques on the daylight operation of a free-space QKD system. The optical parts of the transmitter comprise four laser diodes, a silica-based polarization module, neutral density filters, a telescope, and components for signal synchronization. Real-time data processing was performed with an field-programmable gate array (FPGA) based system. In the receiver, a four-channel single-photon detector (SPD) was installed with a polarization-decoding module for the BB84 protocol. Additional optics for polarization control and filtering systems were used. Both transmitter and receiver were installed above gimbal systems, which allow elevation and azimuth control for beam alignment. FPGA, field programmable gate array; LD, laser diode; Sync. LD, synchronization laser diode; Sync. PD, synchronization photodiode; SPD, single-photon detector; DM, dichroic mirror; HWP, half-wave plate; ND filter, neutral density filter; PBS, polarization beam splitter; BS, beam splitter.

An experimental setup for investigating the effect of filtering techniques on the daylight operation in a free-space QKD system is shown in Fig. 1. We implemented a free-space polarization-based BB84 QKD system. The transmitter unit, normally called Alice, has four single-longitudinal mode vertical-cavity surface-emitting lasers at a lasing wavelength of 785 nm for generating four different polarization states. The temperature of the laser diodes was elaborately controlled in order to make the spectral characteristics of the four laser diodes indistinguishable. Electrical current pulses with a full width at half-maximum (FWHM) of 200 ps were randomly injected into the four lasers at a clock rate of 100 MHz to generate photon pulses with an FWHM of 65 ps, whose intensity level was attenuated into the mean photon number per pulse of 0.1. Our self-developed silica-based chip-integrated polarization module coupled with single-mode fibers (SMFs) was installed for polarization encoding^9,10^. An additional laser source was used at a wavelength of 1550 nm for the system synchronization. The beam leaving the Schmidt-Cassegrain telescope at the transmitter was expanded to a diameter of approximately 30 mm to make its Rayleigh range much longer than the distance of our free-space link.

At the receiver side, Bob, multiple spectral filters were installed to block optical photons for all spectral regions from 200 nm to 2000 nm except for an optical spectral bandwidth of 1 nm for the wavelength of our sources. A combination of a telescope and a spatial filter composed of lenses and a pinhole was used to limit the FOV of the system, which removes the scattered noise photons effectively in the spatial domain. A silicon avalanche photodiode-based four-channel SPD (PerkinElmer SPCM-AQ4C) was used for photon detection with an efficiency of approximately 50% at the wavelength of our sources. The dark count was measured as roughly 300 counts per second for each channel. Noise elimination in the temporal domain and the sifting process for key distillation were conducted at the real-time FPGA data processing system, while the detection counts per second were monitored at the counter. The polarization rotation due to the SMF, connected with our polarization module installed in the transmitter, was compensated at the receiver with a polarization controller unit composed of two quarter-wave plates and one half-wave plate. The transmitter and receiver were deployed on two distant buildings 275 m apart in Electronics and Telecommunication Research Institute (ETRI), South Korea.

**Figure 2:**
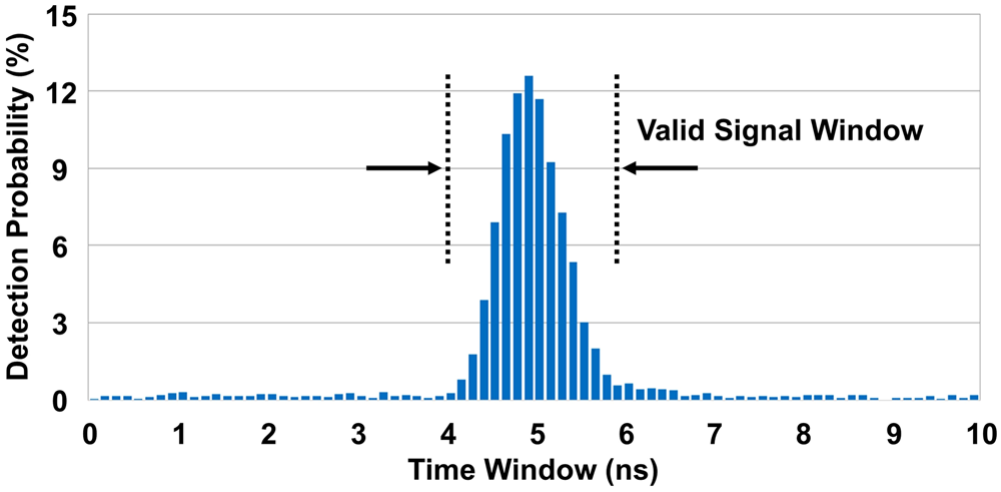
Probability distribution of the detection events of the SPD for QKD operation at a clock rate of 100 MHz at night. It was obtained at the FPGA data processing unit with a timing resolution of 125 ps. The FWHM of the probability distribution was estimated as approximately 750 ps.

Even though the width of the generated optical pulses was approximately 65 ps, the temporal width of the detected quantum signals at the FPGA system was broadened to an FWHM of approximately 750 ps due to SPD response time jitter, clock signal jitter, and other jitters of electronic devices as shown in Fig. 2. Most of our quantum signals were detected within a time window of 1.75 ns. In our experiments, atmospheric turbulences played no role in FHWM broadening effects because quantum signals were transmitted over the short distance of 275 m. Note that slight detection events spread in all temporal regions were caused by spontaneously emitted photons because of a non-negligible DC bias level to the laser diodes, which is an important requirement to eliminate recently reported critical side channel effects of random bit generation with multiple laser diodes^11,12^.

While the superior performance of the QKD system during the night can be easily achieved even without elaborate filtering techniques, the QBER of the system increases significantly during the daytime, possibly much higher than 11%, if there is no adequate noise-filtering system. In addition, the detection count of the SPD can hit its saturation level owing to enormous number of scattered photons from sunlight, which leads to inappropriate operation of the QKD system. Thus, we first set a baseline filtering system so as to maintain the QBER lower than 11%, the minimum requirement for the theoretical security of QKD with one-way post-processing without pre-processing^13^. From the baseline filtering setup, we further improved our system performance stepwise by adopting stronger filtering techniques in the spatial and temporal domains.

Our baseline of the noise elimination techniques was composed of multiple off-the-shelf spectral filters, which allows 1 nm FWHM transmission in the spectral domain, temporal filtering with a valid signal window of 2.5 ns, and a spatial filtering system limiting the FOV as 566 *μ*rad. The aforementioned spectral and spatial filtering system made approximately 10% loss each to our quantum signals, whereas the temporal filtering with a time window of 2.5 ns safely covers all quantum signals as shown in Fig. 2. Experiments were conducted during the night and daytime in order to clearly analyze the effects of noise photons from sunlight. The night QKD experiment was performed on May 24th at 20:00 Korea Standard Time (KST). The daylight QKD experiments were conducted on May 25th and 29th, both of which were bright sunny days and had almost the same level of noise counts from sunlight. All experimental data in daylight were measured within an hour, from 11:30 to 12:30 KST, so as to maintain the similar noise level for the entire experiments. Note that prolonged experiment time is not desirable because the level of noise photons changes even within the daytime.

## Experimental Results

### Performance of QKD during the night and daytime with the baseline filtering system

First, we measured the counting levels of noise photons and quantum signals during the night and daytime with our baseline filtering techniques except for the temporal one for 10 minutes as shown in Table 1. In case of the night, the noise count was mostly attributed to dark counts of the SPD, which are considerably smaller than the signal counts. However, the noise level was increased up to 578,565 counts per second in daylight with a spatial FOV of 566 *μ*rad, which is no longer negligible. It roughly accounts for 1/3 of all detection events including the quantum signals, which highly likely to cause high quantum bit error rate (QBER). Fortunately, a substantial amount of noise counts can be reduced using the temporal filtering technique because noise photons are equally distributed in all temporal regions while quantum signals are detected within a short period of time as shown in Fig. 2.

**Table 1:** Comparison of the average detection counts per second and their standard deviations during the night and daytime each measured for 10 min.

	Noise Mean (Std. Dev.)	Noise + Signal Mean (Std. Dev.)
Night	1,415 (59)	959,956 (20,406)
Daytime	578,565 (12,656)	1,548,087 (48,757)

All represented data are the summation of the detection counts of the four-channel SPD, monitored at the counter. The same filtering techniques were applied for all cases as described in our baseline noise elimination setting except for the temporal one. The noise counts represent the number of detection events without QKD operation, which are solely caused by the scattered noise photons and dark counts of the SPD. The summation of noise and signal counts represents the number of detection events with QKD operation where background noise photons and quantum signals coexist. Std. Dev.: standard deviation.

**Figure 3:**
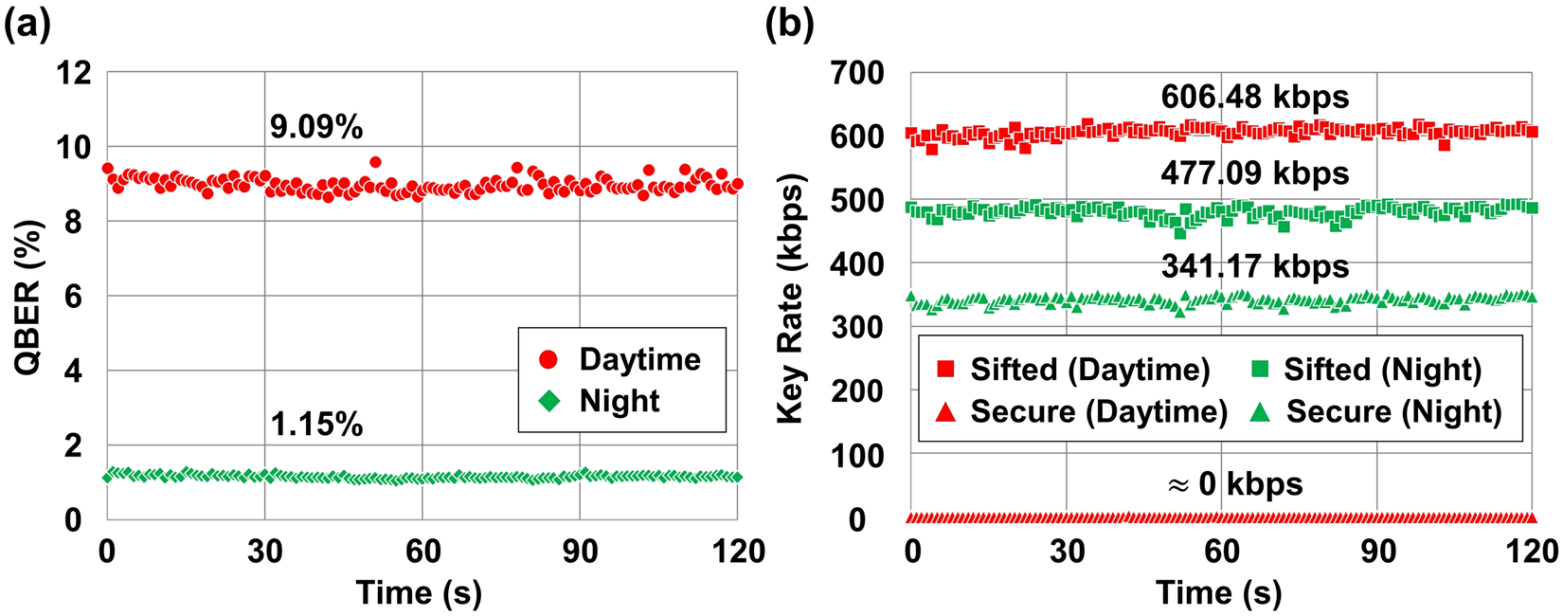
Performance comparison of the QKD system for the daytime and night with the baseline filtering setup. Each performance parameter was recorded per second for 2 min. (**a**) QBER. (**b**) Sifted key rate and secure key rate.

The performances of QBER, sifted key rate, and secure key rate with our baseline filtering system for the night and daytime are shown in Fig. 3. The secure key rate was calculated using the following equation with the assumption of the decoy state method^14–16^.

1 $$R\approx q\{-\eta \mu f({e}_{det}){H}_{2}({e}_{det})+\eta \mu {e}^{-\mu }[1-{H}_{2}({e}_{det})]\},$$

where *q* is the sifting ratio, *μ* is the mean photon number, *η* is the transmittance, *e*_*det*_ is the QBER, *f*(*e*_*det*_) is the error correction coefficient for given *e*_*det*_, and *H*_2_(*e*_*det*_) is the Shannon entropy where ${H}_{2}({e}_{det})=-\,{e}_{det}{\mathrm{log}}_{2}({e}_{det})$ $-\mathrm{(1}-{e}_{det}){\mathrm{log}}_{2}\mathrm{(1}-{e}_{det})$. Here, we used 1.22 for *f*(*e*_*det*_). During the night, the QBER and the secure key rate were measured as 1.15% and 341.17 kbps, respectively as shown in Fig. 3. On the other hand, the QBER was significantly increased up to 9.09% in daylight, which results in no secure keys as shown in Fig. 3(b). Even though the BB84 protocol is known to be able to make non-zero keys if the QBER is smaller than 11%^13^, it should be much lower in order to generate secure keys in practical situations because of error correction efficiency^16^. Thus, our baseline filtering was turned out to be not sufficient for the QKD operation in daylight. Meanwhile, the key rates were recorded with negligible fluctuations in the time domain even though the relative position between the transmitter and the receiver can vary with time in a free-space channel owing to building vibration and sway^17^.

### Performance improvement with further spatial filtering

**Figure 4:**
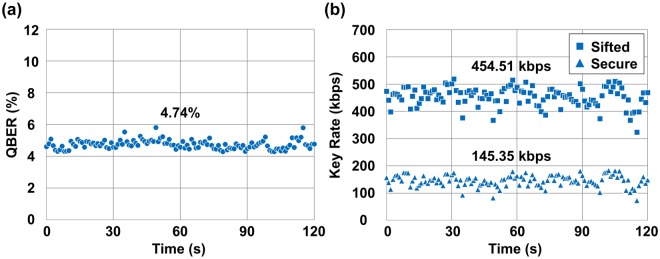
Performance of QKD during the daytime with an FOV of 283 *μ*rad. Each performance parameter was recorded per second for 2 min. (**a**) QBER. (**b**) Sifted key rate and secure key rate.

To reduce the number of noise photons further, we replaced the pinhole used for the spatial filtering system with a smaller one to limit the spatial FOV to 283 *μ*rad. The noise counts reduced to 289,958 per second, which is roughly a half compared to the case with an FOV of 566 *μ*rad. The performances of the QBER and secure key rate are shown in Fig. 4. The QBER was notably reduced to 4.74% from 9.09%, which achieved a secure key rate of 145.35 kbps. It is a considerable improvement compared with the previous case in daylight where no secure keys were generated. Thus, limiting the spatial FOV is an effective way to improve the performance in the daylight operation.

However, a too small FOV may not be beneficial for the performance in terms of the secure key rate because not only the noise photons, but also a fraction of our quantum signals starts to be filtered out as the FOV becomes small. By comparing Fig. 3(b) with Fig. 4(b), one can easily find out that the key rates fluctuate significantly for the FOV of 283 *μ*rad. Quantitatively, the average sifted key rates were estimated as 606,482 bps and 454,514 bps, whereas the standard deviations were calculated as 6,997 bps and 34,261 bps for the cases with FOVs of 566 *μ*rad and 283 *μ*rad, respectively. Even though the sifted key rate is decreased by approximately 25%, the standard deviation shows a five-fold increase under the FOV of 283 *μ*rad, which indicates that the additional spatial limitation with a smaller pinhole makes the key rate fluctuate significantly. This is because a portion of quantum signals is cut off more frequently for a small FOV due to unavoidable vibration of the beam propagation path caused by building vibration and sway^17^, which results in loss of the quantum signals. For transmissions over much longer distances where severe spatial beam fluctuation is inevitable, a beam tracking system with adaptive optics is indispensable in order to alleviate the spatial loss of the quantum signals by compensating the spatial fluctuation of the beam propagation path in real-time. For our case, without the beam tracking system, a spatial limitation less than the FOV of 283 *μ*rad was not desirable because of considerable fluctuations and loss of the quantum signals.

### Performance improvement with further temporal filtering

**Figure 5:**
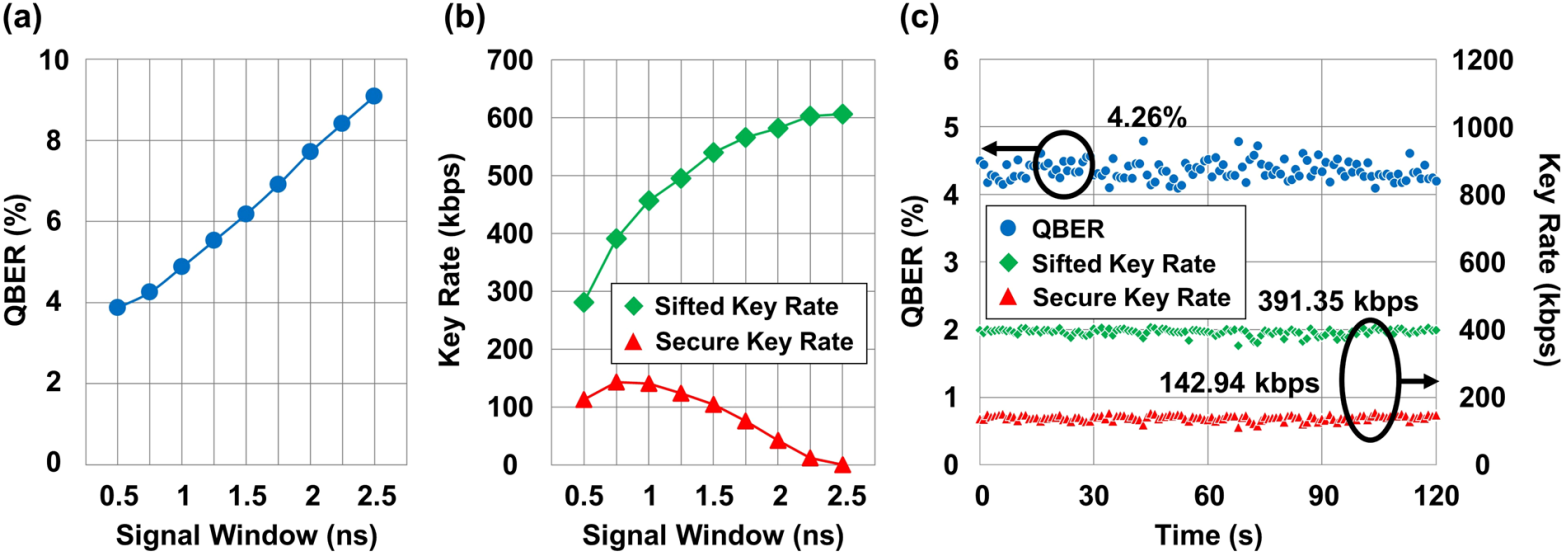
Performance optimization with the temporal filtering technique in daylight. The FOV is fixed at 566 *μ*rad. (**a**) QBER as a function of the valid size of the signal window. (**b**) Sifted and secure key rates as functions of the valid size of the signal window. (**c**) Performance of the QBER and key rates with the optimal signal window size.

Additional temporal filtering from the baseline filtering system was also conducted in the post-processing step in order to improve the performance. We decreased the size of the valid signal window from 2.5 ns to 0.5 ns with a step of 0.25 ns for the case with a spatial FOV of 566 *μ*rad. While the QBER was estimated as 9.09% with a signal window of 2.5 ns, it was decreased to 3.87% with a signal window of 0.5 ns as depicted in Fig. 5(a). However, the number of quantum signals and noise photons decreased also as the size of the signal window became small as shown in Fig. 5(b). In our experiments, the optimal size of the signal window in terms of the secure key rate was 0.75 ns, which achieved a QBER and a secure key rate of 4.26% and 142.94 kbps, respectively. Figure 5(c) presents the results of the performance as a function of time with the optimal signal window.

### Performance optimization with both spatial and temporal filtering

**Figure 6:**
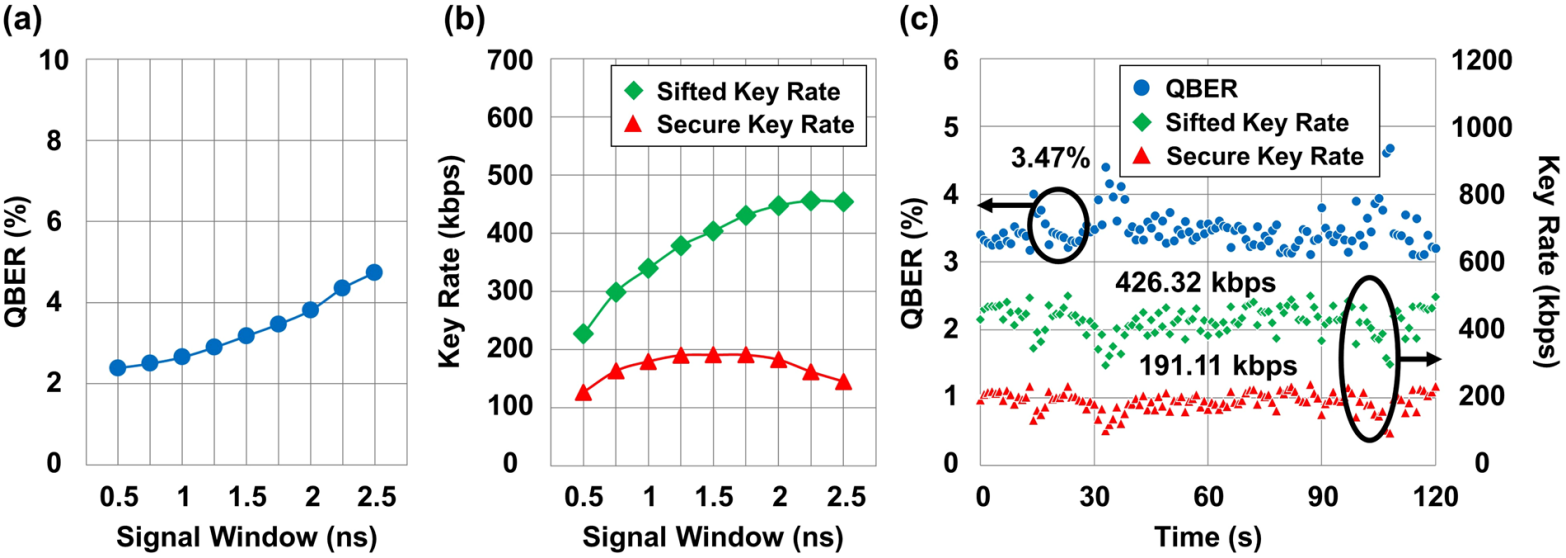
Performance optimization with the temporal filtering technique in daylight. The FOV is fixed at 283 *μ*rad. (**a**) QBER as a function of the valid size of the signal window. (**b**) Sifted and secure key rates as functions of the valid size of the signal window. (**c**) Performance of the QBER and key rates with the optimal signal window size.

Performance optimization using both spatial and temporal filtering techniques was conducted as shown in Fig. 6. In this case, we reduced the size of the signal window in the time domain as discussed in the previous section with an FOV of 283 *μ*rad. The QBER started to decrease from 4.74% to 2.39% as the size was decreased from 2.5 ns to 0.5 ns as shown in Fig. 6(a). Likewise, both sifted key rates and secure key rates decreased with an additional decrease in the signal window due to elimination of quantum signals as shown in Fig. 6(b). Even though the QBER was lowest when the signal window was set to 0.5 ns, the optimal size of the signal window was turned out to be around 1.75 ns, which achieved a secure key rate of 191.11 kbps. Figure 6(c) shows the results of the performance as a function of time at the signal window of 1.75 ns. As discussed with Fig. 4, fluctuations of the key rates increased for the case of an FOV of 283 *μ*rad compared with the case where the FOV was 566 *μ*rad.

As discussed above, the optimal size of the signal window in terms of the secure key rate was quite different for FOVs of 566 *μ*rad and 283 *μ*rad as 0.75 ns and 1.75 ns, respectively. For the case of an FOV of 566 *μ*rad, diminishing the QBER by reducing the size of the signal window was highly important because high QBER causes severe degradation of the secure key rates as described in Eq. 1. That is why decreasing the signal window to 0.75 ns is desirable for performance optimization even though a large amount of quantum signal as well as noise photons is eliminated as shown in Fig. 2. However, in case of an FOV of 283 *μ*rad, the QBER improvement with smaller signal windows is not dramatic because a large amount of noise photons are already eliminated by the spatial filtering technique. Instead, setting an adequate size of the signal window becomes important in order not to sacrifice a large amount of quantum signals, which was estimated to be around 1.75 ns for our experiments.

## Discussion

Among the aforementioned filtering techniques in three different domains, the effect of the spectral filtering is the simplest one to predict. The narrower spectral width of the band pass filter is, the more noise photons are eliminated, which improves the system performance unless quantum signals are cut off in the spectral domain. For our experiments, we used several off-the-shelf BPFs to allow only 1 nm FWHM transmission at our wavelength of 785 nm. Considering the fact that the spectral width of the laser sources is significantly narrower than 1 nm, noise photons can be even further diminished by using a narrower BPF, which will improve the performance of the QKD system^4,6,18^.

The limitation of the spatial FOV should be performed considering the fluctuation of the beam propagation path for the given free-space link. For a short-distance free-space channel like our case, the variation of the relative position between the transmitter and the receiver is mainly caused by building vibration and sway rather than the effects of beam wandering and scintillation^19,20^. Such fluctuations can be compensated with a beam tracking system, commonly consisting of tracking laser sources, position sensors, and fast steering mirrors^1,6^. Fluctuations of the key rates observed in our experiments under an FOV of 283 *μ*rad can be decreased by using a precise and sensitive tracking system. Without precise beam tracking systems, an optimal FOV should be carefully selected considering both beam fluctuations and the level of noise from daylight.

The temporal filtering technique is highly related with the overall system jitter. As discussed in the previous sections, 65 ps FWHM optical pulses were unavoidably broadened due to time jitter of SPD and response time delay of all electronic devices. If one can achieve much smaller system jitter, which results in a sharper detection distribution in the time domain, the temporal filtering technique will be even effective because noise photons can be eliminated without sacrificing quantum signals even for an extremely narrow signal window. Thus, the distribution of the detection events in the time domain should be considered for selecting the optimal size of the signal window.

## Conclusion

In this study, we quantitatively compared the effects of filtering techniques in the spectral, spatial, and temporal domains for noise elimination of a free-space polarization-based BB84 QKD system under the daylight condition. We showed that a QKD system which generates a secure key rate of 341.17 kbps at night could not generate secure keys in daylight although a noise filtering combination of 1 nm spectral BPF, 2.5 ns temporal selection, and spatial FOV of 566 *μ*rad was used. We further demonstrated that a secure key rate of 191.11 kbps can be achieved under the daylight condition using additional filtering techniques with a signal window of 1.75 ns and an FOV of 283 *μ*rad. In addition, we discussed the key parameters and related issues of the performance optimization using filtering techniques in the spectral, spatial, and temporal domains for a free-space QKD system in daylight.

## References

[1] Schmitt-Manderbach T (2007). Experimental demonstration of free-space decoy-state quantum key distribution over 144 km. Phys. Rev. Lett..

[2] Yin J (2017). Satellite-based entanglement distribution over 1200 kilometers. Sci..

[3] Buttler WT (2000). Daylight quantum key distribution over 1.6 km. Phys. Rev. Lett..

[4] Hughes RJ, Nordholt JE, Derkacs D, Peterson CG (2002). Practical free-space quantum key distribution over 10 km in daylight and at night. New J. Phys..

[5] Peloso MP, Gerhardt I, Ho C, Lamas-Linares A, Kurtsiefer C (2009). Daylight operation of a free space, entanglement-based quantum key distribution system. New J. Phys..

[6] Liao S-K (2017). Long-distance free-space quantum key distribution in daylight towards inter-satellite communication. Nat. Photonics.

[7] Dynes JF (2016). Ultra-high bandwidth quantum secured data transmission. Sci. Reports.

[8] Bennet, C. H. Quantum cryptography: Public key distribution and coin tossing. In *Proc. of IEEE Int. C*onf. o*n Comp., Syst. and Signal Proc., Bangalore, India, Dec.* 10–12, 1984 (1984).

[9] Choe, J.-S., Ko, H., Choi, B.-S., Kim, K.-J. & Youn, C. J. Silica planar lightwave circuit based integrated 1 × 4 polarization beam splitter module for free-space bb84 quantum key distribution. *IEEE Photonics J*. (2018).

[10] Choe, J.-S., Ko, H., Choi, B.-S., Kim, K.-J. & Youn, C. J. Integrated polarization beam splitter module for polarization-encoded free-space bb84 qkd. In *Optical Fiber Communication Conference*, Th2A–5 (Optical Society of America, 2018).

[11] Ko H (2017). Critical side channel effects in random bit generation with multiple semiconductor lasers in a polarization-based quantum key distribution system. Opt. Express.

[12] Ko H (2018). High-speed and high-performance polarization-based quantum key distribution system without side channel effects caused by multiple lasers. Photonics Res..

[13] Shor PW, Preskill J (2000). Simple proof of security of the bb84 quantum key distribution protocol. Phys. Rev. Lett..

[14] Hwang W-Y (2003). Quantum key distribution with high loss: toward global secure communication. Phys. Rev. Lett..

[15] Lo H-K, Ma X, Chen K (2005). Decoy state quantum key distribution. Phys. Rev. Lett..

[16] Ma X, Qi B, Zhao Y, Lo H-K (2005). Practical decoy state for quantum key distribution. Phys. Rev. A.

[17] Arnon S (2003). Effects of atmospheric turbulence and building sway on optical wireless-communication systems. Opt. Lett..

[18] Höckel D, Koch L, Martin E, Benson O (2009). Ultranarrow bandwidth spectral filtering for long-range free-space quantum key distribution at daytime. Opt. Lett..

[19] Dios F, Rubio JA, Rodrguez A, Comerón A (2004). Scintillation and beam-wander analysis in an optical ground station-satellite uplink. Appl. Opt..

[20] Andrews, L. C. & Phillips, R. L. *Laser beam propagation through random media*, vol. 152 (SPIE press Bellingham, WA, 2005).

